# Sandflies in an urban area of transmission of visceral leishmaniasis in midwest Brazil

**DOI:** 10.1051/parasite/2016035

**Published:** 2016-09-05

**Authors:** Maria Elizabeth Cavalheiros Dorval, Elisa Teruya Oshiro, Andreia Fernandes Brilhante, Vânia Lúcia Brandão Nunes, Geucira Cristaldo, Manoel Sebastião Costa Lima Júnior, Eunice Aparecida Bianchi Galati

**Affiliations:** 1 Federal University of Mato Grosso do Sul 79070900 Campo Grande MS Brazil; 2 Human Parasitology Laboratory, Anhanguera-Uniderp University 79035740 Campo Grande MS Brazil; 3 Faculty of Public Health, Epidemiology Department, University of São Paulo 01246904 São Paulo SP Brazil; 4 Department of Immunology, Research Center Aggeu Magalhães 50670420 Recife PE Brazil

**Keywords:** *Bichromomyia flaviscutellata*, *Leishmania infantum*, *Lutzomyia longipalpis*, Phlebotomine, Natural infection

## Abstract

The phlebotomine fauna of Campo Grande city, capital of Mato Grosso do Sul state in Brazil, an endemic area for visceral leishmaniasis, has been thoroughly investigated, but all the insect collections were undertaken with automatic light traps. The present study sought to investigate the fauna in this city using Shannon and Disney traps, having human beings and hamsters, respectively, as bait. Both types of traps were installed in forest fragment and peridomiciliary areas in the period from 2007 to 2009. The phlebotomine females were analyzed by PCR for *Leishmania* identification. *Lutzomyia longipalpis* was the only species collected in the peridomiciles and rendered a total of 574 specimens with a 5.2:1 male:female ratio. A total of eight species were attracted to the two traps (one of each type) installed in the forest fragment, including: *Bichromomyia flaviscutellata*, *Evandromyia bourrouli*, *Evandromyia lenti*, *Lutzomyia longipalpis*, *Nyssomyia whitmani*, *Pintomyia christenseni*, *Psathyromyia bigeniculata*, and *Sciopemyia sordellii.* A total of 143 specimens were collected, *Bi. flaviscutellata* accounting for 81% and *Lu. longipalpis* for 1.4% of them. In one female of *Lu. longipalpis* collected in a Disney trap installed in a peridomicile, *Leishmania* (*Leishmania*) *infantum* DNA was found, thus strengthening the hypothesis that the transmission of leishmaniasis is in fact occurring in the anthropic environment.

## Abstract (fr)

La faune des phlébotomes de la ville de Campo Grande, capitale du Mato Grosso do Sul, une zone endémique pour la leishmaniose viscérale, a été soigneusement étudiée, mais toutes les récoltes d’insectes ont été entreprises avec des pièges lumineux automatiques. La présente étude visait à étudier la faune dans cette ville à l’aide de pièges Shannon et Disney, qui utilisent respectivement des êtres humains et des hamsters comme appât. Les deux types de pièges ont été installés dans des fragments de forêt et les zones péridomiciliaires de 2007 à 2009. Les femelles de phlébotomes ont été analysées par PCR pour l’identification des *Leishmania*. *Lutzomyia longipalpis* était la seule espèce recueillie dans les péridomiciles et a produit un total de 574 échantillons avec un ratio mâles/femelles de 5.2/1. Au total, huit espèces ont été attirées par les deux pièges (un de chaque type) installé dans le fragment de forêt : *Bichromomyia flaviscutellata*, *Evandromyia bourrouli*, *Evandromyia lenti*, *Lutzomyia longipalpis*, *Nyssomyia whitmani*, *Pintomyia christenseni*, *Psathyromyia bigeniculata* et *Sciopemyia sordellii.* Un total de 143 échantillons a été collecté, dont *Bi. flaviscutellata* représentait 81 % et *Lu. longipalpis* 1.4 %. Dans une femelle de *Lu. longipalpis* recueillie dans un piège Disney installé dans un péridomicile, de l’ADN de *Leishmania* (*Leishmania*) *infantum* a été trouvé, renforçant ainsi l’hypothèse que la transmission de la leishmaniose se produit dans l’environnement anthropique.

## Introduction

Campo Grande city, the capital of Mato Grosso do Sul state, is an endemic area for visceral leishmaniasis (VL). The notification of cases began in the early 2000s, after the first studies of the phlebotomine fauna revealed the presence of *Lutzomyia longipalpis*, the main vector of the VL agent, at the end of the 1990s. Since then, several studies have been conducted in Campo Grande sampling forest and anthropic environments to better understand the diversity of species, as well as aspects of insect behavior [2, 22–24]. However, in all these studies, the phlebotomine fauna was sampled using only automatic light traps.

To understand the natural history of the parasites transmitted by these insects, and to incriminate a particular species of phlebotomine as a *Leishmania* vector, beyond the repeated isolation of the parasite in the phlebotomine, one also needs to consider aspects of the diversity of the species in the area in which the disease occurs, their relative abundance, the determination of the degree of anthropophily and zoophily of the species, and their ecological distribution [11, 16].

In order to gain knowledge on the sand flies of Campo Grande city, the present study sought to investigate the phlebotomine fauna using two other types of traps, i.e. Shannon and modified Disney traps [7], with a view to ascertaining the respective attractiveness of human beings and rodents to these insects, both in forest and peridomiciliary environments.

## Materials and methods

### Study area

The municipality of Campo Grande, with an area of 8,092.951 km^2^ and an estimated population of 843,120, is located in the central region of Mato Grosso do Sul, covering 2.27% of the total area of the state. The City Hall is situated at 20°26′34″ S and 54°38′47″ W [15].

In accordance with Köppen’s classification [17], the predominant climate is of the rainy, tropical savannah type, subtype AW, characterized by irregular annual distribution of rainfall, with a well-defined dry season during the coldest months of the year and a rainy period during the summer months. The average annual temperature is about 23 °C, December being the hottest month at approximately 25 °C and June the coldest with an 18 °C average [15].

### Collections of phlebotomines

The collections of phlebotomines were carried out from 2007 to 2009 in three areas of the city, one in a residual forest, Parque Rita Vieira (20°29′35.49″ S; 54°34′21.67″ W) and two in peridomiciles of residential neighborhoods: one in the Jockey Club (20°33′29.29″ S; 54°36′04.31″ W) and another in Jardim Leblon (20°29′12.22″ S; 54°39′12.22″ W). According to the Center for the Control of Zoonotic Diseases of the Health Secretariat of Campo Grande, the zones where Parque Rita Vieira and the Jockey Club are situated are classified as areas of sporadic transmission, and Jardim Leblon an area of moderate transmission [35].

Trapping in Shannon traps was undertaken quarterly to investigate natural infection by flagellates, in the two peridomiciles and inside the forest, from 6:00 pm to 10:00 pm by two people. The traps were illuminated with cold white light generated by a 12 V battery. The insects were collected in polyethylene flasks which were maintained in a clay and moist plaster recipient until their examination at the Human Parasitology Laboratory of the Federal University of Mato Grosso do Sul (UFMS).

For the identification of the species and search for flagellates, the females were immobilized and the dissection undertaken on a slide containing a drop of sterile saline to expose the digestive track and spermathecae under a stereoscopic microscope. Examination of the gut and identification of the sand fly species were undertaken on an optical microscope at a magnification of 400×.

The male specimens collected were clarified and mounted on slides. All the phlebotomines were identified in accordance with Galati’s key [12].

For the investigation of animal attractiveness, about 50 m from the Shannon trap, a modified Disney trap [7], having a hamster (*Mesocricetus auratus*) as bait, was installed inside the forest and in the peridomicile of the residences. The cage containing the hamster was maintained at about 20 cm from the ground. Each sentinel animal was changed at three-monthly intervals or when it was observed that its general state had deteriorated.

The animals were kept in polyethylene cages, in accordance with the recommendations based on population density (Institute of Laboratory Animal Resources, 1996), with a sawdust bedding of *Pinnus* sp. and received plentiful water and commercial feed Nuvilab CR-1^®^ (Nuvital, Curitiba, PR, Brazil). Monthly, the cages were cleaned, food replaced and the animals examined to detect alterations compatible with leishmaniasis. The phlebotomines adhering to the traps were collected on these occasions for later identification, as described above.

Authorization for this kind of capture was obtained from the Ethics Committee for the Use of Animals of UFMS (CEUA-UFMS) Protocol No. 154/2007.

### Molecular analysis of *Leishmania* strains

DNA extraction was performed after maceration of the insects in 20 μL lysis buffer (50 mM NaCl, 10 mM EDTA, pH 8.0, 50 mM Tris-HCL, pH 7.4, Triton X100 1%, and 10 mM DTT). This was followed by three cycles of freezing (with liquid nitrogen) and thawing (60 °C). This macerate was incubated for 1 h at 60 °C and for another 3 h at 60 °C with the addition of 1 μL of proteinase K (20 mg/mL), 80 μL of lysis buffer, and 1% of Triton X100 per insect. After the incubation, the sample was centrifuged for 10 min at 12,000 *g* and the supernatant collected was added to 0.1 volume of sodium acetate (3 M, pH 5.2) and two volumes of frozen absolute ethanol and this was maintained overnight at −20 °C. The material was again centrifuged at 4 °C for 10 minutes at 13,000 *g*; the precipitate was washed with 70% ethanol, and after being dried, resuspended in 20 μL of distilled H_2_O.

Polymerase chain reaction (PCR) conditions were selected according to Lima-Júnior et al. [19] and standardized with the control DNA supplied by the Laboratory of Leishmaniasis of the René Rachou Research Center (Belo Horizonte, Brazil): *Le.* (*Le.*) *infantum* (MHOM/BR/74/PP/75), *Le.* (*Viannia*) *braziliensis* (MHOM/BR/75/M2903), and *Le.* (*Le.*) *amazonensis* (IPLA/ BR/67/PH8).

## Results

During the study period, in both types of traps installed in peridomiciles and the forest, a total of 717 phlebotomine specimens were collected: 485 (67.6%) of them males and 232 (32.4%) females, with a male:female sex ratio of 2.1:1.0 (Table 1). The phlebotomines belonged to seven genera and eight species: *Bichromomyia flaviscutellata* (Mangabeira, 1942), *Evandromyia bourrouli* (Barretto & Coutinho, 1941), *Evandromyia lenti* (Mangabeira, 1938), *Lutzomyia longipalpis* (Lutz & Neiva, 1912), *Nyssomyia whitmani* (Antunes & Coutinho, 1939), *Pintomyia christenseni* (Young & Duncan, 1994), *Psathyromyia bigeniculata* (Floch & Abonnenc, 1941), and *Sciopemyia sordellii* (Shannon & Del Ponte, 1927).

**Table 1.** Distribution of phlebotomines collected in the peridomiciles and forest fragment by sex and trap, Campo Grande, Mato Grosso do Sul, Midwest Brazil, 2007–2009.

Trap	Shannon		Subtotal		Disney		Subtotal		Total
	Sex								
Species	♂	♀	♂♀	♂/♀	♂	♀	♂♀	♂/♀	♂♀
*Bi. flaviscutellata*	1	–	1	–	40	75	115	0.53	116
*Ev. bourrouli*	–	–	–	–	9	6	15	1.5	15
*Ev. lenti*	–	2	2	–	–	–	–	–	2
*Lu. longipalpis*	78	15	93	5.20	355	128	483	2.77	576
*Ny. whitmani*	1	2	3	0.50	–	–	–	–	3
*Pi. christenseni*	–	–	–	–	–	1	1	1.00	1
*Pa. bigeniculata*	1	1	2	1.00	–	–	–	–	2
*Sc. sordellii*	–	2	2	–	–	–	–	–	2
Total	81	22	103	3.68	404	210	614	1.92	717

*Lu. longipalpis* was the only species collected in the peridomiciliary environment, both in the Shannon and Disney traps; the male:female ratio being 5.2:1.0 and 2.8:1.0, respectively.

Among the 143 specimens of eight species collected in the forest, *Bi. flaviscutellata* accounted for 81.1% followed by *Ev. bourrouli* at 11.3%. The other species were collected in small numbers. *Bi. flaviscutellata* was almost exclusively collected in the Disney trap, with a male:female ratio of 0.5:1.0 and *Ev. bourrouli* only in this trap, the male:female ratio being of 1.5:1.0. *Lu. longipalpis* and *Pi. christenseni* were also only collected in the Disney trap. On the other hand, *Ev. lenti*, *Ny. whitmani*, *Pa. bigeniculata*, and *Sc. sordellii* occurred only in the Shannon trap (Tables 1 and 2).

**Table 2.** Distribution of the phlebotomines collected in Shannon and modified Disney traps in peridomiciles and forest fragment, Campo Grande, Mato Grosso do Sul, Midwest Brazil, 2007–2009.

Species	Environment/trap													
	Peridomicile Shannon		Peridomicile Disney		Subtotal		Forest Shannon		Forest Disney		Subtotal		Total	
	*n*	%	*n*	%	*n*	%	*n*	%	*n*	%	*n*	%	*n*	%
*Bi. flaviscutellata*	0	0.0	0	0.0	0.0	0.0	1	0.1	115	16.1	116	16.2	116	16.2
*Ev. bourrouli*	0	0.0	0	0.0	0.0	0.0	0	0.0	15	2.1	15	2.1	15	2.1
*Ev. lenti*	0	0.0	0	0.0	0.0	0.0	2	0.3	0	0.0	2	0.3	2	0.3
*Lu. longipalpis*	93	12.9	481	67.1	574	80.0	0	0.0	2	0.3	2	0.3	576	80.3
*Ny. whitmani*	0	0.0	0	0.0	0.0	0.0	3	0.4	0	0.0	3	0.4	3	0.4
*Pi. christenseni*	0	0.0	0	0.0	0.0	0.0	0	0.0	1	0.1	1	0.1	1	0.1
*Pa. bigeniculata*	0	0.0	0	0.0	0.0	0.0	2	0.3	0	0.0	2	0.3	2	0.3
*Sc. sordellii*	0	0.0	0	0.0	0.0	0.0	2	0.3	0	0.0	2	0.3	2	0.3
Total	93	12.9	481	67.1	574	80.0	10	1.1	133	18.3	143	20.0	717	100.0

During the four hours of collection (6:00 pm–10:00 pm) in Shannon traps, *Lu. longipalpis* was present throughout, though in greater numbers during the two last hours (Table 3).

**Table 3.** Distribution of phlebotomines collected in a Shannon trap by hour in peridomiciles and forest fragment, Campo Grande, Mato Grosso do Sul, Midwest Brazil, 2007–2009.

Species	Time									
	6–7 pm		7–8 pm		8–9 pm		9–10 pm		Total	
	*n*	%	*n*	%	*n*	%	*n*	%	*n*	%
*Bi. flaviscutellata*	0	0.0	0	0.0	0	0.0	1	1.0	1	1.0
*Ev. lenti*	0	0.0	0	0.0	1	1.0	1	1.0	2	1.9
*Lu. longipalpis*	8	7.8	13	12.6	36	35.0	36	35.0	93	90.3
*Ny. whitmani*	2	1.9	1	0.9	0	0.0	0	0.0	3	3.0
*Pa. bigeniculata*	0	0.0	1	0.9	0	0.0	1	1.0	2	1.9
*Sc. sordellii*	0	0.0	0	0.0	2	1.9	0	0.0	2	1.9
Total	10	9.7	15	14.4	39	37.9	39	37.9	103	100.0

Flagellates were observed in the midguts of one specimen of *Lu. longipalpis* collected in the Disney trap installed in the peridomicile; the parasite being identified as *Leishmania* (*Leishmania*) *infantum* by PCR. The sentinel animals were kept under observation in the vivarium of the Human Parasitology Laboratory of UFMS for a year but presented no manifestations compatible with leishmaniasis.

## Discussion

All the phlebotomines collected in the present study had been reported among the 31 species which comprise the fauna previously registered in Campo Grande. *Lu. longipalpis* had already been reported as the most frequent species, occurring in high densities in residential areas, mainly those with animal shelters such as chicken coops near residences. However, in the forests, even when domestic animals, which may act as population amplifiers for this sand fly, were present in their surroundings, the species was not predominant and an increase of the richness and diversity of species has been observed [22–24, 26, 27, 33]. This was also observed in this present study, which shows that *Lu. longipalpis* has adapted to the peridomicile and is independent of the forest for its reproduction and maintenance.

*Pa. bigeniculata* is registered here for the first time in Campo Grande, but one should bear in mind that this taxon was recently resurrected from being a synonym of *Pa. shannoni* [30].

The Shannon trap is useful for the capture of anthropophilic species because as human beings are present during the collections, they act as bait attracting the insects both by their odor and release of CO_2_, thus reflecting the feeding habits of the species [1, 13, 28]. However, the luminous stimulus of the trap should be borne in mind as well as the fact that the trap may be functioning as an interception barrier to the flight of insects. Thus, the presence of *Lu. longipalpis* in this kind of trap may be influenced either by the attraction of the human beings or by the light and may also be due to the trap acting as an interception barrier. In this case, the release of the male pheromone would attract other conspecific males and females forming aggregates (leks) of specimens [10] on the ecotope (trap) and thus explain the high male:female ratio (5.2:1.0). On the other hand, although males were captured predominantly in the Disney trap, this ratio here was of 2.8:1.0. This fact seems to indicate a greater attractiveness to rodents than to human beings for *Lu. longipalpis* females, though Oliveira et al. [25] have reported that 66.4% of the female specimens of this species fed on human blood.

The collection of *Sc. sordellii*, known to take its blood meals on frogs, exclusively by the Shannon trap seems to be explained either by its luminous attractiveness or by its acting as an interception barrier.

In a study undertaken in Campo Grande by Oliveira et al. [25], *Lu. longipalpis* showed an eclectic feeding habit; however, among the samples tested, no positive result was observed for rodents. Thus, the collection of this species in Disney traps in the peridomicile with a male:female ratio below that observed for the Shannon traps reinforces its opportunist habits, as demonstrated in other studies regarding the diversity of feeding sources. This is an aspect relevant to the epidemiology and the understanding of the mechanisms of transmission of visceral leishmaniasis [21, 25, 29].

*Lu. longipalpis*, active throughout this study’s four-hour collection period, the highest frequencies occurring between 8.00 pm and 10.00 pm, was the only species collected in the peridomiciles and was rarely observed in the forest. These facts reinforce the hypothesis that the VL agent is transmitted in the anthropic environment, where human beings coexist with domestic and synanthropic animals under conditions favorable to the abundance of this phlebotomine.

*Bichromomyia flaviscutellata*, a phlebotomine of rodentophilic habits [9], has already been found in the urban areas of Campo Grande and Bonito [4, 22–24] as well as in forested areas of Bela Vista [9] and of the Bodoquena Plateau [14], all of them in Mato Grosso do Sul state. The presence of this species, the principal vector of *Le. amazonensis* in forest fragments adjacent to residences, is worrying and demonstrates its gradual adaptation to the human environment as has already been observed in other areas [3]. It also serves as an alert as to the possibility of outbreaks of cutaneous leishmaniasis (CL) due to the imbalances caused by anthropic modifications of phlebotomine habitats. In Mato Grosso do Sul, infections by *Le. amazonensis* have already been reported in human beings, cats, and phlebotomines [4, 8, 34].

Although some phlebotomines show specificity as regards the species of *Leishmania* which they transmit, it should be mentioned that both natural and experimental infection of *Lu. longipalpis* by *Le*. *amazonensis* has been observed [6, 31] and its vectorial competence in the transmission of this parasite to the hamster has been demonstrated by Sherlock [32].

In Mato Grosso do Sul, an area in which the distribution of *Le. amazonensis*, *Le. infantum*, and *Le.* (*Viannia*) sp*.* overlaps, *Lu. longipalpis* has been found naturally infected by these three parasites and has been classified as a permissive vector [31].

The other species presented low frequency, being found exclusively in the interior of the forest fragment, though attention should be drawn to the presence of *Ny. whitmani*, a vector of *Le. braziliensis*, which does not eliminate the possibility of this species being associated with the transmission of CL in the region. As well as being provenly anthropophilic [5], it has demonstrated its ability to adapt to modified human environments [14].

Thus, this study emphasizes the importance of *Lu. longipalpis* in the epidemiology of visceral leishmaniasis in Campo Grande. Further, with the strategy used for the insect collections it was possible to demonstrate that *Bi. flaviscutellata* is the most frequent species in the forest fragment where it was almost exclusively attracted to the Disney trap. Its role as the main vector of *Le. amazonensis*, which has rodents as its main hosts, is well established and although this sand fly was absent in the peridomicile, synanthropic rodents might serve as a bridge for this parasite between the forest and the peridomicile where these animals can be bitten by *Lu. longipalpis*, thus introducing and maintaining the parasite in a peridomiciliary cycle [18, 20].

On the basis of the results obtained in this study, and the rodentophilic behavior of *Lu. longipalpis* and *Bi. flaviscutellata*, studies on the role of synanthropic rodents as reservoirs of *Leishmania* species and their participation in the leishmaniasis transmission cycle in Campo Grande are necessary.

## Conflict of interest

The authors declare no conflict of interest in relation with this paper.
